# *N,N,N′,N′*-tetrakis(2-pyridylmethyl)ethylenediamine, a zinc chelator, inhibits biofilm and hyphal formation in *Trichosporon asahii*

**DOI:** 10.1186/s13104-020-04990-x

**Published:** 2020-03-10

**Authors:** Sanae Kurakado, Ryota Chiba, Chisato Sato, Yasuhiko Matsumoto, Takashi Sugita

**Affiliations:** Department of Microbiology, Meiji Pharmaceutical University, 2-522-1 Noshio, Kiyose, Tokyo 204-8588 Japan

**Keywords:** Biofilm, Chelator, *N*,*N*,*N′,N′*-tetrakis(2-pyridylmethyl)ethylenediamine, *Trichosporon asahii*

## Abstract

**Objective:**

*Trichosporon asahii* is the major causative fungus of disseminated or deep-seated trichosporonosis and forms a biofilm on medical devices. Biofilm formation leads to antifungal drug resistance, so biofilm-related infections are relatively difficult to treat and infected devices often require surgical removal. Therefore, prevention of biofilm formation is important in clinical settings. In this study, to identify metal cations that affect biofilm formation, we evaluated the effects of cation chelators on biofilm formation in *T. asahii*.

**Results:**

We evaluated the effect of cation chelators on biofilm formation, since microorganisms must assimilate essential nutrients from their hosts to form and maintain biofilms. The inhibition by *N*,*N*,*N′,N′*-tetrakis(2-pyridylmethyl)ethylenediamine (TPEN) was greater than those by other cation chelators, such as deferoxamine, triethylenetetramine, and ethylenediaminetetraacetic acid. The inhibitory effect of TPEN was suppressed by the addition of zinc. TPEN also inhibited *T. asahii* hyphal formation, which is related to biofilm formation, and the inhibition was suppressed by the addition of zinc. These results suggest that zinc is essential for biofilm formation and hyphal formation. Thus, zinc chelators have the potential to be developed into a new treatment for biofilm-related infection caused by *T. asahii*.

## Introduction

Pathogenic microorganisms adapt to host environments and cause various infectious diseases. Many of the pathogens, which cause systemic infections through the bloodstream, form biofilms on the surface of medical devices [1, 2]. Biofilm infections can have disastrous consequences, as biofilm cells embedded with extracellular matrix are usually resistant to both antimicrobial agents and host immune systems [3]. The cells themselves also change expression levels of resistance-related genes. Finally, dispersion of biofilms leads to systemic infections. When biofilm infections occur, surgical removal of the infected device is required. Therefore, biofilm formation on the surface of medical devices is a problem in clinical settings.

*Trichosporon asahii* is a major causative pathogenic fungus of deep-seated trichosporonosis and can form biofilms on various medical device substrates [4]. Deep-seated trichosporonosis develops in immunocompromised patients, specifically in those with hematological malignancies accompanied by neutropenia, such as leukemia. *T. asahii* is the third most common etiological agent of fungemia, after *Candida* species and *Cryptococcus* species [5, 6]. *Candida albicans*, a representative dimorphic fungus, forms biofilms on the surface of medical devices [7, 8]. *T. asahii* is also a dimorphic fungus with a yeast form, hyphal form, and arthroconidia that disarticulate from hyphae [9]. Because of the commonality in morphological transition, between the two organisms, the literature on *C. albicans*-infections provides important background to the present study. The clinical outcome of disseminated trichosporonosis is poorer than that of disseminated candidiasis caused by *Candida* species [5]; the mortality rate is over 70% [10, 11]. Recently, disseminated trichosporonosis has frequently been reported in immunocompromised patients on echinocandin treatment, because *Trichosporon* species are highly resistant to the echinocandins [12, 13]. Infections caused by *Trichosporon* species have become more increasing since the use of echinocandins, which are first-line antifungal agents used in emergency and intensive care medicine. Therefore, preventive methods are required to inhibit biofilm formation, which contributes to deep-seated trichosporonosis [4].

We previously found that the expression of *Pra1* and *Zrt1* genes involved in zinc utilization in *C. albicans* was greater in biofilms than in planktonic cells [14]. The *pra1* and *zrt1* gene-deficient strains exhibited decreased abilities to form biofilms and also hyphae, which are essential for biofilm formation [14]. Moreover, zinc addition caused increased biofilm formation, and zinc depletion by a chelator inhibited biofilm formation in *C. albicans* [14]. These findings suggest that zinc is a key metal for biofilm formation in *C. albicans*. However, the importance of zinc for biofilm formation in *T. asahii* remains poorly understood.

In this study, we evaluated the effects of cation chelators on biofilm formation by *T*. *asahii*. *N*,*N*,*N′,N′*-tetrakis(2-pyridylmethyl)ethylenediamine (TPEN), a cation chelator, effectively inhibited biofilm formation by *T*. *asahii* and reduced hyphal formation. The inhibitory effects of TPEN were eliminated by the addition of excess amounts of zinc. Our findings suggest that zinc is a key metal for biofilm formation by *T. asahii* and that TPEN is a potent inhibitor of biofilm formation.

## Main text

### Materials and methods

#### Reagents

Four chelators were examined in this study: TPEN (Tokyo Chemical Industry Co., Ltd., Tokyo, Japan), deferoxamine (DFO; Abcam, Cambridge, UK), and triethylenetetramine (TETA; Nacalai Tesque, Kyoto, Japan), which predominately chelate Zn^2+^, Fe^2+^ and Cu^2+^, respectively, and ethylenediaminetetraacetic acid (EDTA; Nippon Gene Co., Ltd, Tokyo, Japan), which predominantly chelates divalent cations such as Mg^2+^ and Ca^2+^. Tested final concentrations were [0, 0.01, 0.1, 1, 10, 100 μM] for TPEN, [0, 50, 500, 5000, 50,000 μM] for DFO and [0, 5, 50, 500, 5000 μM] for TETA and EDTA. TPEN was dissolved in DMSO. DFO, TETA and EDTA were dissolved in sterile water. All stock solutions were stored at − 30 °C until use.

#### *T. asahii* culture

The stock strain, *T. asahii* MPU 105, which is highly able to form biofilms, was selected from the MPU culture collection, and the strain was isolated from the blood of patient. The strain was routinely grown on Sabouraud dextrose agar plates at 27 °C.

#### Biofilm formation

Biofilms formed from cells grown in flat-bottomed, 96-well microtiter plates. Standardized cell suspensions (A_630_ = 0.1 in RPMI 1640-plus-MOPS medium, pH 7.0) were seeded into each well. The plates were incubated without shaking at 37 °C for 1 h, and then the supernatants were removed and the cells were washed with PBS. Next, fresh medium was added, followed by incubation without shaking at 37 °C, and then the supernatants were removed after 24 h and replaced with fresh medium. Planktonic cells were removed after an additional 24 h incubation and the wells were washed with PBS. Semiquantitative evaluation of biofilm formation was performed using the XTT reduction assay. Absorbance was measured at wavelengths of 492 and 630 nm.

#### Microscopic analysis

*T*. *asahii* cells (A_630_ = 0.1) were added to RPMI 1640-plus-MOPS medium with each concentration of TPEN and ZnSO_4_ in 96-well plates and incubated without shaking at 37 °C for 48 h. Controls (no TPEN or ZnSO_4_) included the same amount of solvent as the other samples. Morphological changes were microscopically evaluated.

#### Statistical analysis

The significance of differences between groups was calculated using a Student’s *t* test. *P *< 0.05 was considered a statistically significant difference.

### Results

**Fig. 1:**
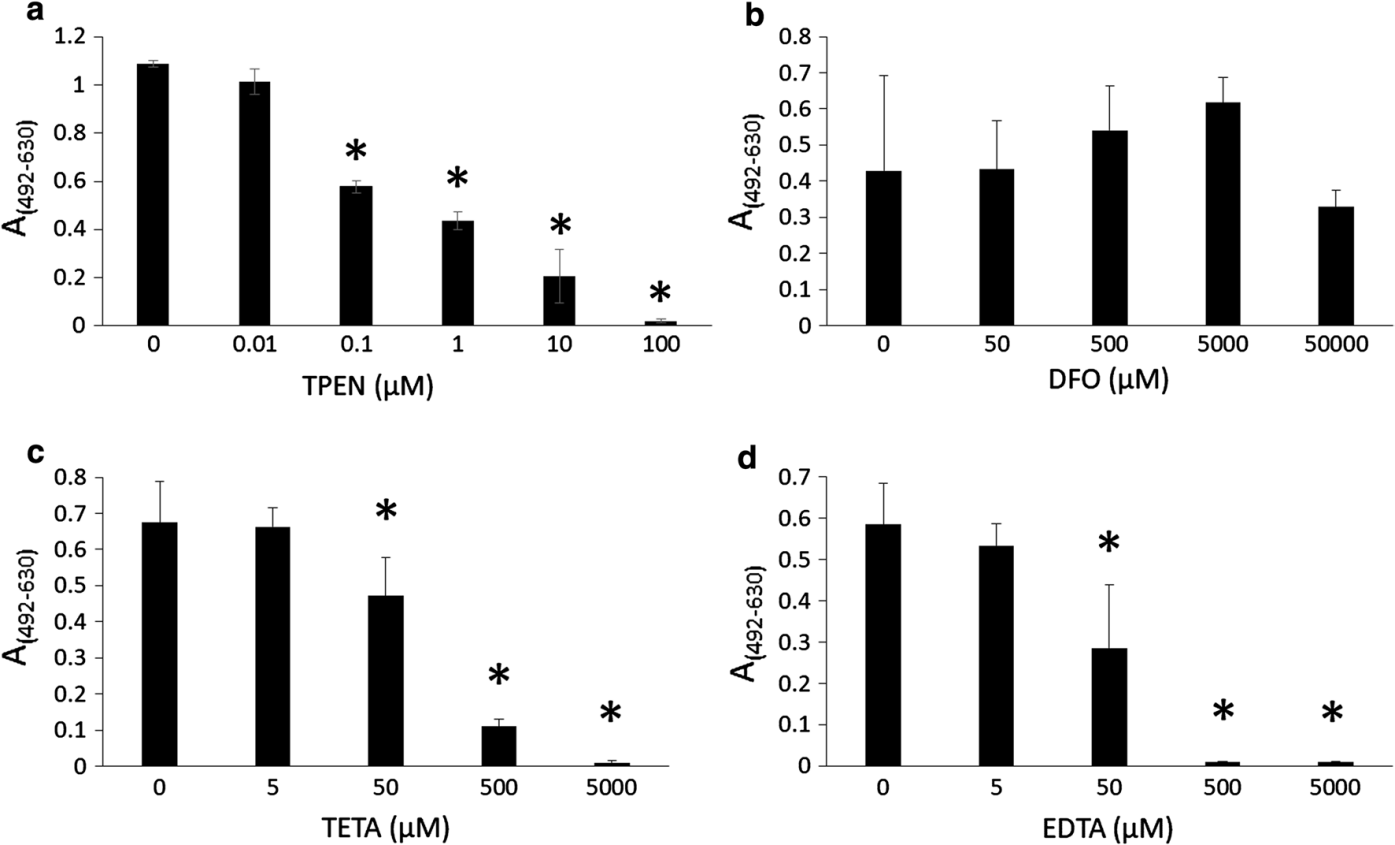
Biofilm formation in the presence of divalent chelators Biofilms were incubated in RPMI-plus-MOPS medium with the following cation chelators: **a***N*,*N*,*N′,N′*-tetrakis(2-pyridylmethyl)ethylenediamine, **b** deferoxamine, **c** triethylenetetramine, and **d** ethylenediaminetetraacetic acid. Controls included the same amount of each solvent without chelators. Biofilm formation was measured using the XTT reduction assay. Measurements were performed four times under each condition, and data are expressed as means ± standard deviations. *P < 0.05 compared to controls

We tested whether biofilm formation by *T. asahii* was inhibited by the cation chelators TPEN, DFO, TETA, and EDTA. TPEN, EDTA, and TETA reduced biofilm formation in a concentration-dependent manner (Fig. 1). DFO did not obviously inhibit biofilm growth at 50 mM (Fig. 1). IC_50_ values (the concentration of a compound required for the half inhibition of biofilm formation) of TPEN, TETA, and EDTA were 0.17 µM, 117 µM, and 47 µM, respectively. TPEN was the most effective cation chelator to inhibit biofilm formation by *T. asahii*.

**Fig. 2:**
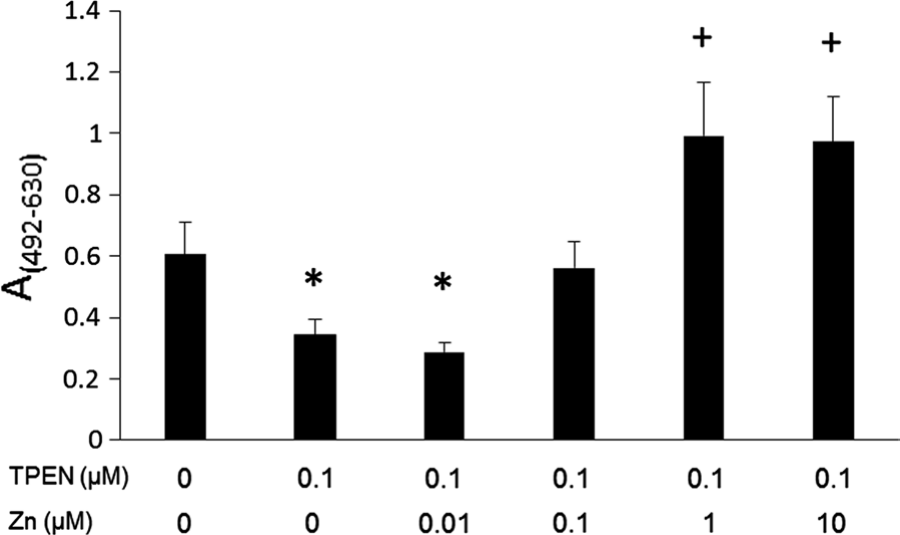
Biofilm inhibition by *N*,*N*,*N′,N′*-tetrakis(2-pyridylmethyl)ethylenediamine (TPEN) is abrogated by zinc Biofilms were incubated in medium including 0.1 μM TPEN and 0.01–10 μM zinc, respectively. Controls (no TPEN, no ZnSO4) included the same amount of each solvent. Biofilm formation was measured using the XTT reduction assay. Measurements were performed four times under each condition, and data are expressed as means ± standard deviations. *P < 0.05 relative to controls (decrease). +*P*<0.05 relative to DMSO controls (increase)

TPEN has been used as a zinc chelator in several studies [15–17]. Therefore, we tested the effect of zinc on biofilm formation by *T. asahii*. Adding 1 to 10 µM of zinc increased biofilm formation, while high concentration of zinc decreased biofilm formation (Additional file 1: Figure S1). Zinc suppressed the inhibitory effects of TPEN on biofilm formation by *T. asahii* (Fig. 2). Moreover, the addition of excess amounts of zinc led to an increase in biofilm formation by *T. asahii* (Fig. 2). These results suggest that zinc is an important metal for biofilm formation by *T. asahii*. We examined whether the inhibitory effect of TPEN was reversible by removing TPEN from the medium. After 1 h or 24 h treatment with TPEN, TPEN was removed by changing the medium (Additional file 2: Figure S2a). Biofilm formation by *T. asahii* was increased by removing TPEN (Additional file 2: Figure S2b). The effect of zinc addition was also observed in this assay (Additional file 2: Figure S2b). These results indicate that TPEN treatment did not kill all *T. asahii* cells and that the inhibitory effect of TPEN was reversible.

**Fig. 3:**
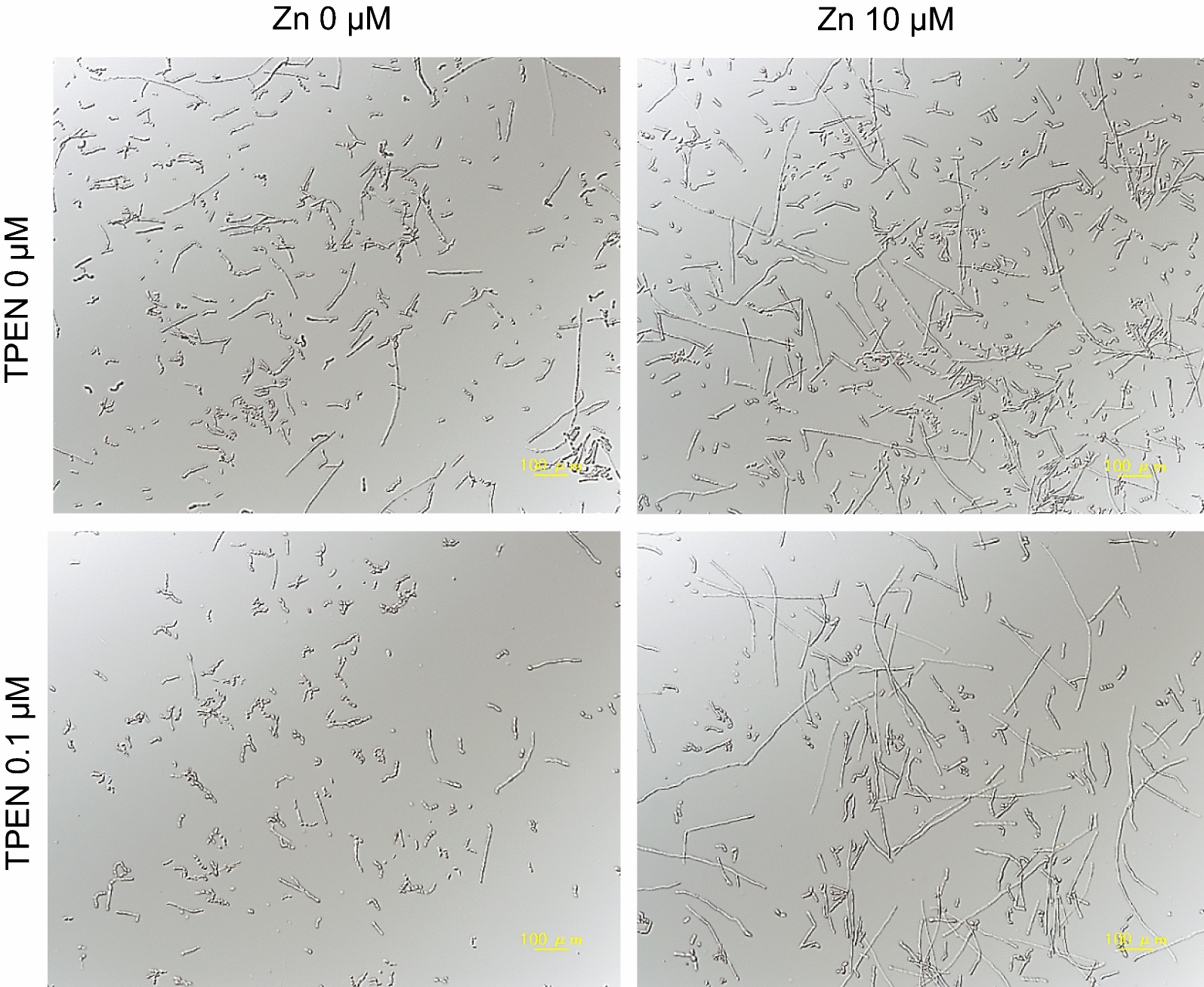
Effect of *N*,*N*,*N′,N′*-tetrakis(2-pyridylmethyl)ethylenediamine (TPEN) and zinc on morphological transitions in *T*. *asahii T*. *asahii* was cultured in RPMI-plus-MOPS medium containing TPEN, zinc, or TPEN plus zinc, at 37 °C. Controls (no TPEN, no ZnSO4) included the same amount of each solvent. Cells were observed under a light microscope after 48 h incubation

We next examined morphological changes induced by TPEN. Control cells formed hyphae and arthroconidia. In the presence of 0.1 μM TPEN, hyphal formation decreased, whereas zinc addition induced hyphal elongation (Fig. 3). These results suggest that zinc regulated hyphal formation of *T*. *asahii*.

### Discussion

In this study, the cation chelator TPEN inhibited biofilm formation and hyphal elongation in *T*. *asahii*. Zinc triggered hyphal elongation and enhanced biofilm formation. Only morphological transition in response to TPEN or zinc was observed in planktonic cells in this study. Direct observation of biofilm structure changes instigated by TPEN or zinc is an area for future research. Our findings suggest that *T. asahii* uses zinc as an environmental indicator.

In *C*. *albicans*, zinc is important in terms of both biofilm development and maintenance. The expression of genes related to zinc acquisition increases in biofilm cells of *C*. *albicans* [14, 18]. Our previous report suggested that *C. albicans* biofilm formation is regulated by zinc and that TPEN inhibits biofilm formation [14]. Depletion of divalent cations, such as iron, calcium, and magnesium, inhibit biofilm formation by *Aspergillus fumigatus* and *Cryptococcus neoformans* [19, 20]. In a murine model of invasive pulmonary aspergillosis caused by pathogenic fungus *Aspergillus fumigatus*, TPEN improved survival [16]. In vivo experiments to evaluate the inhibitory effect of TPEN on biofilm formation by *T. asahii* are areas for future research.

Zinc also promotes biofilm formation by the pathogenic bacterium *Streptococcus pneumoniae* [21]. Derivatives of 2-aminobenzimidazole inhibit biofilm development in methicillin-resistant *S*. *aureus*, vancomycin-resistant *Enterococcus faecium* and *S*. *epidermidis*, by chelating zinc [22]. In *S*. *aureus*, zinc activates intercellular adhesion during biofilm formation [23]. Thus, zinc may play a pivotal role in biofilm formation in both eukaryotes and prokaryotes. Therefore, cation chelators effectively prevent and treat biofilm-related infections.

In conclusion, zinc chelating has the potential to inhibit biofilm formation by *T. asahii*. Although further research is essential, TPEN could potentially be applied to catheters to prevent biofilm formation. The information may contribute to the prevention of biofilm formation by *T. asahii* on the surface of medical devices.

## Limitations

The results of this study are limited to in vitro conditions and a single *T. asahii* strain. Further in vivo studies are required to clarify whether zinc deficiency through cation chelation would be effective against *T. asahii* biofilm infections. Also, studies using a wide variety and number of strains are recommended.

## Supplementary information

**Additional file 1: Fig. S1.** Biofilm formation in the presence of zinc, and absence of chelators Biofilms were incubated in medium including 1–500 μM ZnSO_4_. Controls (no ZnSO4) included the same amount of each solvent. Biofilm formation was measured using an XTT reduction assay. Measurements were performed four times under each condition, and data are expressed as means ± standard deviations. *P < 0.05 relative to controls (decrease). +*P*<0.05 relative to DMSO controls (increase).

**Additional file 2: Fig. S2.** Enhancement of biofilm formation by zinc. **a** Experimental scheme. Biofilms were grown for 1 h or 24 h in medium containing *N*,*N*,*N′,N′*-tetrakis(2-pyridylmethyl)ethylenediamine (TPEN), after which the medium was removed and replaced with TPEN-free medium or TPEN-free medium containing zinc. We included medium, dimethyl sulfoxide (DMSO), and TPEN controls. TPEN was added to 0.1 μM and zinc to 1 or 10 μM. Biofilm formation was measured using the XTT reduction assay. **b** Measurements were performed four times under each condition, and data are expressed as means ± standard deviations. *P < 0.05 relative to DMSO controls (decrease). +*P*<0.05 relative to DMSO controls (increase). ++*P *< 0.05 relative to both DMSO and medium controls (increase).

## Data Availability

All datasets generated during this study are included in this published article and its supplementary information file.

## Abbreviations

DFO: Deferoxamine
EDTA: Ethylenediaminetetraacetic acid
TETA: Triethylenetetramine
TPEN: *N*,*N*,*N′,N′*-tetrakis(2-pyridylmethyl)ethylenediamine

## References

[1] Costerton JW, Stewart PS, Greenberg EP (1999). Bacterial biofilms: a common cause of persistent infections. Science.

[2] Ramage G, Martínez JP, López-Ribot JL (2006). *Candida* biofilms on implanted biomaterials: a clinically significant problem. FEMS Yeast Res.

[3] Lohse MB, Gulati M, Johnson AD, Nobile CJ (2018). Development and regulation of single- and multi-species *Candida albicans* biofilms. Nat Rev Microbiol.

[4] Di Bonaventura G, Pompilio A, Picciani C, Iezzi M, D’Antonio D, Piccolomini R (2006). Biofilm formation by the emerging fungal pathogen *Trichosporon* *asahii*: development, architecture, and antifungal resistance. Antimicrob Agents Chemother.

[5] Yamamoto M, Takakura S, Hotta G, Matsumura Y, Matsushima A, Nagao M (2013). Clinical characteristics and risk factors of non-*Candida* fungaemia. BMC Infect Dis.

[6] Lin SY, Lu PL, Tan BH, Chakrabarti A, Wu UI, Yang JH (2019). The epidemiology of non-*Candida* yeast isolated from blood: The Asia Surveillance Study. Mycoses.

[7] Cardinal E, Braunstein EM, Capello WN, Heck DA (1996). *Candida albicans* infection of prosthetic joints. Orthopedics.

[8] Kojic EM, Darouiche RO (2004). *Candida* infections of medical devices. Clin Microbiol Rev.

[9] Colombo AL, Padovan AC, Chaves GM (2011). Current knowledge of *Trichosporon* spp. and trichosporonosis. Clin Microbiol Rev.

[10] Girmenia C, Pagano L, Martino B, D’Antonio D, Fanci R, Specchia G (2005). GIMEMA infection program. Invasive infections caused by *Trichosporon* species and *Geotrichum capitatum* in patients with hematological malignancies: a retrospective multicenter study from Italy and review of the literature. J Clin Microbiol.

[11] Suzuki K, Nakase K, Kyo T, Kohara T, Sugawara Y, Shibazaki T (2010). Fatal *Trichosporon* fungemia in patients with hematologic malignancies. Eur J Haematol.

[12] Matsue K, Uryu H, Koseki M, Asada N, Takeuchi M (2006). Breakthrough trichosporonosis in patients with hematologic malignancies receiving micafungin. Clin Infect Dis.

[13] Go SE, Lee KJ, Kim Y, Choi JK, Kim YJ, Lee DG (2018). Catheter-related *Trichosporon* *asahii* bloodstream infection in a neutropenic patient with myelodysplastic syndrome. Infect Chemother.

[14] Kurakado S, Arai R, Sugita T (2018). Association of the hypha-related protein Pra1 and zinc transporter Zrt1 with biofilm formation by the pathogenic yeast *Candida albicans*. Microbiol Immunol.

[15] Kawamata T, Horie T, Matsunami M, Sasaki M, Ohsumi Y (2017). Zinc starvation induces autophagy in yeast. J Biol Chem.

[16] Laskaris P, Atrouni A, Calera JA, d’Enfert C, Munier-Lehmann H, Cavaillon JM (2016). Administration of zinc chelators improves survival of mice infected with *Aspergillus* *fumigatus* both in monotherapy and in combination with caspofungin. Antimicrob Agents Chemother.

[17] Saini S, Bharati K, Shaha C, Mukhopadhyay CK (2017). Zinc depletion promotes apoptosis-like death in drug-sensitive and antimony-resistance *Leishmania donovani*. Sci Rep.

[18] Nobile CJ, Fox EP, Nett JE, Sorrells TR, Mitrovich QM, Hernday AD (2012). A recently evolved transcriptional network controls biofilm development in *Candida albicans*. Cell.

[19] Nazik H, Penner JC, Ferreira JA, Haagensen JA, Cohen K, Spormann AM (2015). Effects of iron chelators on the formation and development of *Aspergillus fumigatus* biofilm. Erratum in: Antimicrob Agents Chemother 2015;59:7160. Antimicrob Agents Chemother.

[20] Robertson EJ, Wolf JM, Casadevall A (2012). EDTA inhibits biofilm formation, extracellular vesicular secretion, and shedding of the capsular polysaccharide glucuronoxylomannan by *Cryptococcus neoformans*. Appl Environ Microbiol.

[21] Brown LR, Caulkins RC, Schartel TE, Rosch JW, Honsa ES, Schultz-Cherry S (2017). Increased zinc availability enhances initial aggregation and biofilm formation of *Streptococcus pneumoniae*. Front Cell Infect Microbiol.

[22] Rogers SA, Huigens RW, Melander C (2009). A 2-aminobenzimidazole that inhibits and disperses gram-positive biofilms through a zinc-dependent mechanism. J Am Chem Soc.

[23] Formosa-Dague C, Speziale P, Foster TJ, Geoghegan JA, Dufrêne YF (2016). Zinc-dependent mechanical properties of *Staphylococcus aureus* biofilm-forming surface protein SasG. Proc Natl Acad Sci USA.

