# Supplementary figures and images for: N,N,N′,N′-tetrakis(2-pyridylmethyl)ethylenediamine, a zinc chelator, inhibits biofilm and hyphal formation in Trichosporon asahii
Source: BMC Res Notes. 2020 Mar 10;13:142. doi: 10.1186/s13104-020-04990-x (PMC7063706; doi:10.1186/s13104-020-04990-x)

## Slide 1
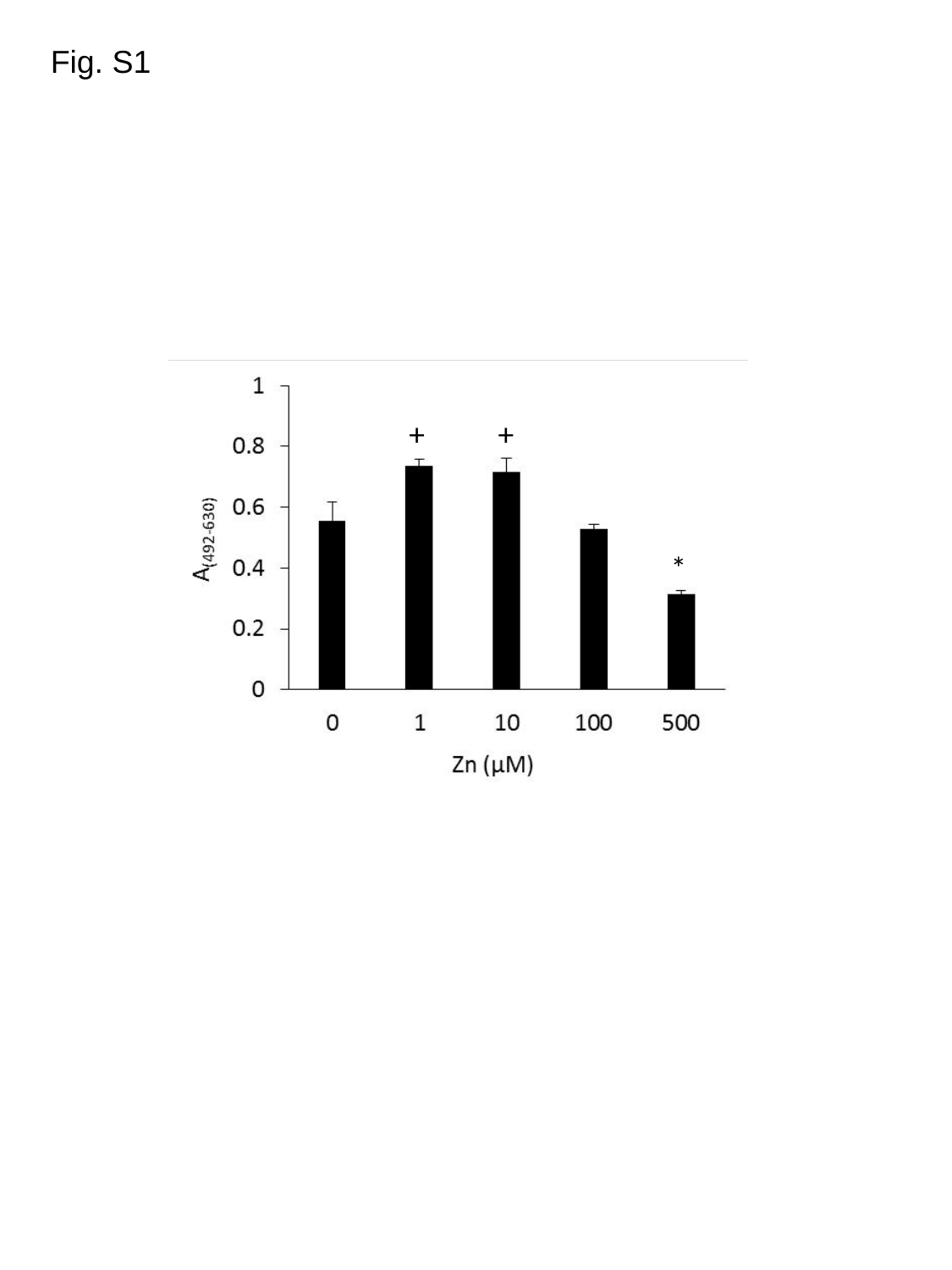

Fig. S1
+
+
*

Supplement: Supplementary file 1 — Additional file 1: Fig. S1. Biofilm formation in the presence of zinc, and absence of chelators Biofilms were incubated in medium including 1–500 μM ZnSO4. Controls (no ZnSO4) included the same amount of each solvent. Biofilm formation was measured using an XTT reduction assay. Measurements were performed four times under each condition, and data are expressed as means ± standard deviations. *P < 0.05 relative to controls (decrease). +P<0.05 relative to DMSO controls (increase). [file 13104_2020_4990_MOESM1_ESM.pptx]

## Slide 1
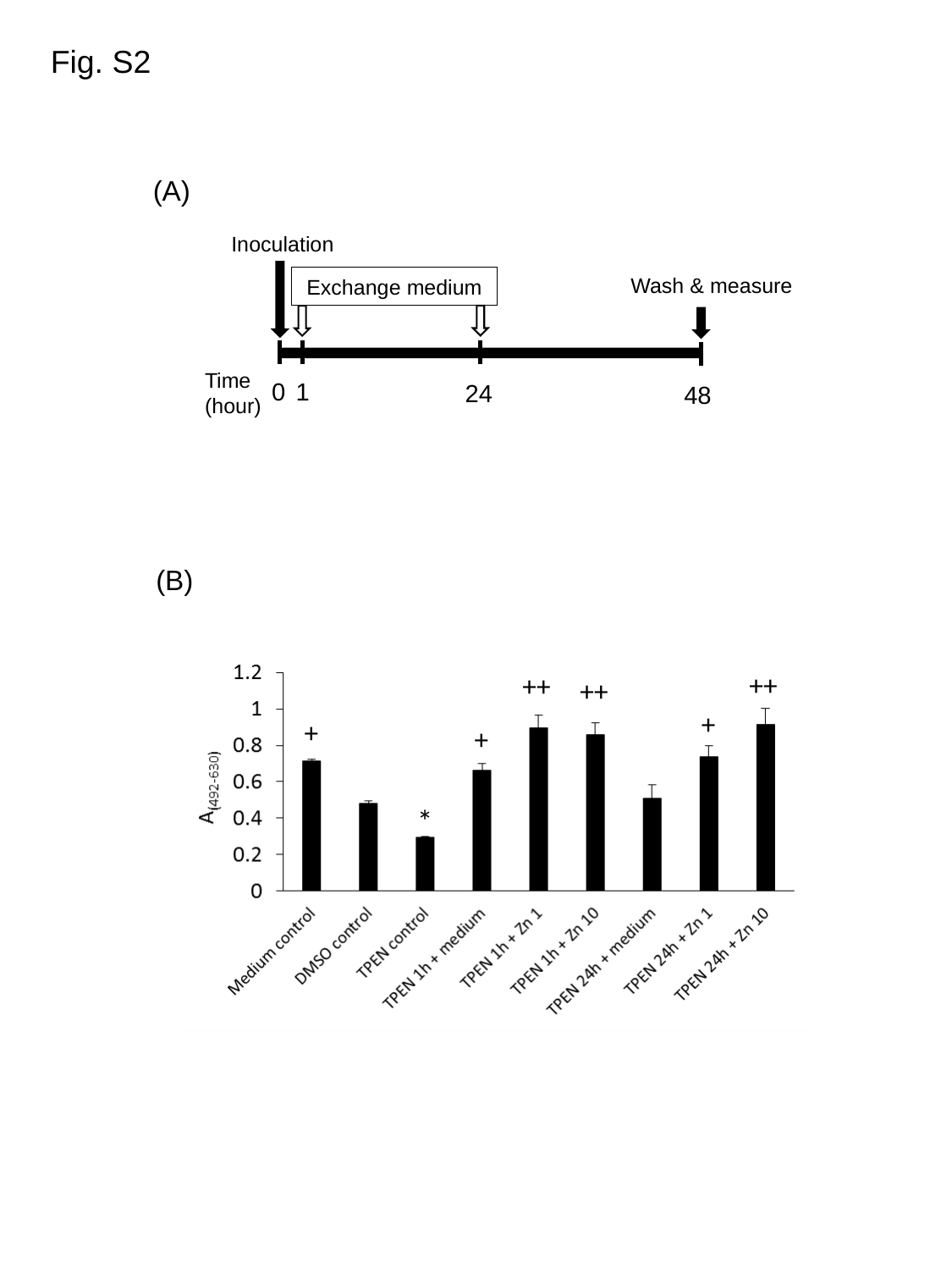

Fig. S2
(A)
Inoculation
Wash & measure
Exchange medium
Time (hour)
0
1
24
48
(B)
++
++
++
+
+
+
*

Supplement: Supplementary file 2 — Additional file 2: Fig. S2. Enhancement of biofilm formation by zinc. a Experimental scheme. Biofilms were grown for 1 h or 24 h in medium containing N,N,N′,N′-tetrakis(2-pyridylmethyl)ethylenediamine (TPEN), after which the medium was removed and replaced with TPEN-free medium or TPEN-free medium containing zinc. We included medium, dimethyl sulfoxide (DMSO), and TPEN controls. TPEN was added to 0.1 μM and zinc to 1 or 10 μM. Biofilm formation was measured using the XTT reduction assay. b Measurements were performed four times under each condition, and data are expressed as means ± standard deviations. *P < 0.05 relative to DMSO controls (decrease). +P<0.05 relative to DMSO controls (increase). ++P < 0.05 relative to both DMSO and medium controls (increase). [file 13104_2020_4990_MOESM2_ESM.pptx]
